# Prevalence of Depression, Anxiety, and Perceived Stress in Postpartum Mexican Women during the COVID-19 Lockdown

**DOI:** 10.3390/ijerph18094627

**Published:** 2021-04-27

**Authors:** Blanca Vianey Suárez-Rico, Guadalupe Estrada-Gutierrez, Maribel Sánchez-Martínez, Otilia Perichart-Perera, Carolina Rodríguez-Hernández, Carla González-Leyva, Erika Osorio-Valencia, Arturo Cardona-Pérez, Addy Cecilia Helguera-Repetto, Salvador Espino y Sosa, Mario Solis-Paredes, Enrique Reyes-Muñoz

**Affiliations:** 1 Community Intervention Research Branch, National Institute of Perinatology, Ministry of Health, Mexico City 11000, Mexico; blancasuarezrico@gmail.com; 2 Research Division, National Institute of Perinatology, Ministry of Health, Mexico City 11000, Mexico; gpestrad@gmail.com; 3 Department of Inmunobiochemistry, National Institute of Perinatology, Ministry of Health, Mexico City 11000, Mexico; maribel71sm@yahoo.com.mx (M.S.-M.); ceciliahelguera@yahoo.com.mx (A.C.H.-R.); 4 Department of Nutrition and Bioprograming, National Institute of Perinatology, Ministry of Health, Mexico City 11000, Mexico; otiliaperichart@inper.gob.mx (O.P.-P.); carolina.rdz94@gmail.com (C.R.-H.); carlapaty90@hotmail.com (C.G.-L.); 5 Department of Developmental Neurobiology, National Institute of Perinatology, Ministry of Health, Mexico City 11000, Mexico; erikaosorio4@hotmail.com; 6 General Direction, National Institute of Perinatology, Ministry of Health, Mexico City 11000, Mexico; acardonadr@gmail.com; 7 Clinical Research Branch, National Institute of Perinatology, Ministry of Health, Mexico City 11000, Mexico; salvadorespino@gmail.com; 8 Department of Genetics, National Institute of Perinatology, Ministry of Health, Mexico City 11000, Mexico; juan.mario.sp@gmail.com; 9 Coordination of Gynecological and Perinatal Endocrinology, National Institute of Perinatology, Ministry of Health, Mexico City 11000, Mexico

**Keywords:** Edinburgh Postnatal Depression Scale, postpartum, anxiety, perceived stress, COVID-19, lockdown

## Abstract

The COVID-19 lockdown represents a new challenge for mental health researchers and clinical practitioners. This cross-sectional study aimed to investigate the prevalence of depression, anxiety, and perceived stress in postpartum Mexican women. The study included 293, 4–12-week postpartum women over the age of 18. The Edinburgh Postpartum Depression Scale (EPDS), Trait-State Trait Anxiety Inventory (T-STAI), and Ten Perceived Stress Scale (PSS-10), which are all questionnaires validated for the Mexican population, were applied using a web-based online survey. Prevalence and 95% confidence intervals (CIs) were calculated. The mean ± standard deviation (SD) of the maternal age was 29.9 ± 6.3 years; the EPDS score: 11 ± 6, T-STAI score: 41.7 ± 12.3, and PSS-10 score: 17.1 ± 7. The prevalence (95% CI) of the postpartum depression symptoms was 39.2% (34–45%), trait anxiety symptoms were found among 46.1% (32–43%) of the participants, and moderate and high perceived stress were in 58% (52–64) and 10.9% (7.8–15) of the participants, respectively. The prevalence of depressive symptoms, generalized anxiety, and perceived stress was higher among postpartum Mexican women during the COVID-19 outbreak than before the lockdown. Our findings highlight the importance of monitoring perinatal mental health during pandemics and the need to design effective psychologic interventions for these patients.

## 1. Introduction

In December 2019, Wuhan, a city in Hubei Province in China, became the center of the emergence of the coronavirus disease 2019 (COVID-19), which is caused by severe acute respiratory syndrome coronavirus 2 (SARS-CoV-2) [1]. As of date, the global cumulative number of cases reached 108.2 million with over 2.3 million deaths since the start of the pandemic [2]. In Mexico, the first wave of COVID-19 occurred from March to July 2020, and as of February 2021, there were 2 million cases [3]. According to the national register of COVID-19 in Mexico, there were 31,253 cases of women with pregnancy/puerperium under follow-up due to COVID-19 infection, 33.6% (*n* = 10,505) had tested positive as of December 2020, of which 205 have died, with a fatality rate of 1.93% and a maternal mortality rate of 10.1 per 100,000 live births. The states with the highest number of positive COVID-19 cases were Mexico City with 1668, Nuevo León with 746, and Guanajuato with 745 [4].

The rapid transmission of COVID-19, high fatality rates in vulnerable populations, lack of effective treatments and vaccines, and mass quarantine measures have led to common mental health problems, such as fear, anxiety, depression, and sleep problems [5].

As a result of the social restrictions, lockdowns, and the reorganization of health systems, the indirect effects of COVID-19 have been evident, even in well-resourced countries, such as Singapore [6]. Hence, in low-income countries, the impact of the containment and preparedness policies on maternal and newborn health could be more pronounced.

Previous studies on the effects of lockdowns on mental health and psychological well-being reported a high prevalence of psychological symptoms of distress [7,8,9]. These stressors include a longer quarantine duration, infection fears, frustration, boredom, inadequate supplies, inadequate information, financial loss, and stigma [7]. A recent meta-analysis described the prevalence of mental health problems during the COVID-19 pandemic in general populations; the overall pooled prevalence of depression, anxiety, distress, and insomnia was 31.4%, 31.9%, 41.1%, and 37.9%, respectively [10].

Physical distancing guidelines have impeded access to social support across populations; it may be acutely felt by pregnant and postpartum women, especially after birth, when caregiving and other social support are needed by the family [11,12,13]. The psychological and social consequences of lockdown can be devastating for high-risk populations, such as pregnant women [14]. The prevalence rates of anxiety, depression, psychological distress, and insomnia among pregnant women during the COVID-19 pandemic have been documented to be as high as 37% (95% confidence interval (CI): 25–49%), 31% (95% CI: 20–42%), 70% (95% CI: 60–79%), and 49% (95% CI: 46–52%), respectively [15].

It has often been thought that pregnancy is protective against the development of depression, primarily because of the lower suicide rate during pregnancy and during the 2 years after giving birth [16]. In reality, the postpartum period was found to be a period of increased risk for the development of major depression symptoms [17].

The physiological and psychological processes experienced in the first weeks after delivery affect the mental health of the mother and increase the risk of postpartum depression [18]. Postpartum depression generally occurs within 4 to 6 weeks after childbirth, and it includes symptoms that are similar to a major depressive disorder [19,20]. Postpartum depression has negative consequences for both mothers, their infants, and their children (up to 3 years of age). It has a significant impact on the mother’s psychological health, quality of life, and interactions with their infant, partner, and relatives. Chronic maternal depression increases the risk of developing milder depression in children and lowers the quality of the home environment; it also results in decreased maternal sensitivity and caregiving. The risks are greater for children in low-income populations [20]. The postpartum depression prevalence rates prior to the COVID-19 pandemic varied between countries, ranging from 6.9–12.9% in high-income countries to more than 20% in some low- or middle-income countries [21].

Previous studies have reported that the prevalence of postpartum mental health disorders, such as depression and anxiety, during the COVID-19 pandemic ranged from 15 to 44% [15,18,22,23,24,25,26]. According to a recent systematic review, few studies have investigated the prevalence of anxiety in the postpartum period [15]. Stepowicz et al. reported a prevalence of 34.8% of high anxiety levels among pregnant and postpartum women and a prevalence of 43.3% of high-stress levels among North American women [27].

There is a lack of information about the prevalence of mental health problems in postpartum Mexican women during the COVID-19 pandemic. Thus, the present study aimed to investigate the prevalence of depression, anxiety, and perceived stress in postpartum Mexican women during the COVID-19 pandemic in a tertiary level hospital.

## 2. Materials and Methods

### 2.1. Study Setting

A cross-sectional study using a web-based online survey was conducted in Mexico City. The study protocol followed the Declaration of Helsinki Ethical Principles for Medical Research Involving Human Subjects. The participants provided their signed informed consent before being included in this work. The study was approved by the Ethics and Research Internal Review Board of the Instituto Nacional de Perinatología in Mexico City (2020-1-32).

### 2.2. Subjects and Procedures

The postpartum women who fulfilled the inclusion criteria were contacted by phone. The purpose of the study was explained to them, and, if they were accepted to participate, their email addresses were requested. Those who agreed to participate in the study received an email with a written consent form and a button to click to access the Google Forms survey. All the questions in the survey were mandatory to avoid the possibility of missing data. Three days after sending the initial email, the women who did not complete the survey were contacted by phone to remind them of the invitation. A second reminder was made one week after the initial email. The women that did not respond two weeks after the first email were considered to have declined the invitation to participate. Data were collected between August and September 2020. Informed consent was obtained by asking all the participants to click a button at the beginning of the online survey to acknowledge their consent.

Demographic and clinical characteristics were obtained from the participants’ clinical records. Women aged >18 years with a single birth, 4 to 12 weeks postpartum, and the ability to read and write Spanish were included.

The exclusion criteria were: stillbirth, women who did not answer the initial telephone call or complete the survey, and those with pre-existing psychiatric disorders (e.g., major depressive disorder, anxiety) based on a medical record review.

Information on sociodemographic characteristics, including maternal age, relationship status, education level, the result of a SARS-CoV-2 test, chronic diseases, parity, and adverse perinatal outcome, was also collected. The adverse perinatal outcome included one or more of the following: preterm birth, preeclampsia, diabetes, obstetric hemorrhage, obstetric hysterectomy, and placenta previa.

All the participants had a universal screening for SARS-CoV-2 using a nasopharyngeal swab via real-time quantitative polymerase chain reaction (RT-qPCR) at the moment of admission for delivery or 24 h before delivery in women that were scheduled for a Cesarean section.

### 2.3. Primary Outcomes and Psychological Questionnaires

The primary outcome was to report the prevalence of depression, anxiety, and stress among postpartum Mexican women. Postpartum depression was evaluated using the Edinburgh Postnatal Depression Scale (EPDS) [28]. The EPDS is a 10-item self-report questionnaire with possible scores ranging from 0 to 30; it is applied to screen for depressive symptoms in the postnatal period. Previously, it has been validated for use in the Mexican population [29,30]. Postpartum depression was defined as an EPDS score of ≥13. The reliability of the EPDS scale was excellent (Cronbach’s alpha = 0.90).

We assessed the anxiety level using the State-Trait Anxiety Inventory (STAI) [31], which is a self-report questionnaire that was standardized and validated for the Mexican population [32] and is confirmed using two separate self-rating scales that are used to measure two different dimensions of anxiety. The Trait Anxiety (T-STAI) scale consists of 20 statements in which individuals are asked to describe how they generally feel. The State Anxiety (S-STAI) scale also consists of 20 statements that require the respondents to know how they feel when applying the scale. The range of scores for each scale is 20–80. Anxiety was defined as a cutoff score of >40 points on the T-STAI scale, which was recommended to detect clinically relevant symptoms of anxiety during postpartum [33]. In our study, the Cronbach’s alpha was 0.93 for the T-STAI scale.

The Ten Perceived Stress Scale PSS-10 [34] is a self-report scale of 10 items measuring the degree to which people perceive their lives to be stressful. Respondents are asked how often they have found their lives to be unpredictable, uncontrollable, and overloaded in the previous month. The 10 items are assessed on a five-point Likert scale with the following response categories: never, rarely, sometimes, fairly often, and very often (scored from 0 to 4). Total scores range between 0 and 40, with higher scores indicating more perceived stress. In the present study, perceived stress was classified as low (0–13), moderate (14–26), or high (27–40), as suggested by others [35,36]. The PSS-10 scale had good reliability (Cronbach’s alpha = 0.89).

### 2.4. Sample Size

The required sample size was 257 women to find a postpartum depression prevalence of 40% with a 95% CI and a 6% error. Our study sample consisted of 293 women that met the inclusion criteria.

### 2.5. Statistical Analysis

Statistical analysis was performed using the Statistical Package for Social Science Software (SPSS 24, IBM, Chicago, IL, USA). Continuous variables were expressed as mean ± standard deviation (SD); categorical variables were reported as the frequency and proportions. Prevalence was calculated with a 95% CI. Pearson or Spearman correlation tests were used based on the type of variable to explore the correlations between each of the sociodemographic and clinical factors and the EPDS, STAI, and PSS-10 scores. The chi-square test was used to evaluate the differences in the proportions. The cutoff for statistical significance was set at *p* < 0.05.

## 3. Results

### 3.1. Characteristics of the Study Population

During the study period, 851 deliveries were attended at our institution, of these women, 425 were excluded because of stillbirth (*n* = 8), multiple gestations (*n* = 42), maternal age <18 years (*n* = 78), previous diagnosis of anxiety or depression (*n* = 17), and they did not answer the initial telephone call (*n* = 280). A total of 426 participants fulfilled the inclusion criteria and were invited to answer the online survey; of these, 293 women answered the survey (68.8%) and were included in the analysis.

The sociodemographic characteristics are shown in Table 1. Most of the women were between the ages of 18 and 34 and were married and unemployed. The most frequent level of schooling was high school.

The gestational age at the pregnancy resolution was 38.2 ± 2.2 weeks. The newborn weight was 2882 ± 631 g. Moreover, 34.5% of the women had complications during pregnancy; the most frequent complications were premature birth and preeclampsia. The participants were tested for SARS-CoV-2; *n* = 54 (18.4%) of the women tested positive; of these, 49 (90.7%) were asymptomatic and 6 (9.3%) had mild symptoms (Table 2).

### 3.2. Psychological Questionnaires’ Outcomes

The mean ± SD of the total scores for EPDS, T-STAI, and PSS-10 were 11 ± 6, 41.7 ± 12.3, and 17.1 ± 7, respectively. The prevalence of depression, trait anxiety, and perceived stress are shown in Table 3.

There was a significant positive correlation between the total scores for PSS-10, T-STAI, and EPDS. There was a significant correlation between the positive SARS-CoV-2 test and the total EPDS score. There was a negative correlation between maternal age and the total T-STAI and EPDS scores. There was no correlation between the other sociodemographic factors and the total scores for PSS-10, T-STAI, and EDPS (Table 4).

There were no statistical differences in the prevalence of depression (28% versus 42%, *p* = 0.06), trait anxiety (39% versus 51%, *p* = 0.12), moderate perceived stress (54% versus 59%, *p* = 0.56), and high perceived stress (9% versus 11%, *p* = 0.56) between the women with a positive SARS-CoV-2 test and those with a negative test.

## 4. Discussion

This is the first web-based cross-sectional study that has aimed to assess the prevalence of the mental health status of postpartum Mexican women faced with an unprecedented COVID-19 pandemic. The survey was distributed from August to September 2020, beyond the peak of the first wave of the pandemic in Mexico. The overall prevalence of significant depressive symptoms in our study was about 39.2%, which was higher than the global prevalence of postpartum depression described before the COVID-19 lockdown, which ranged from 14 to 20% among healthy mothers without a prior history of depression [37]; moreover, the prevalence found in our study was three times higher than the prevalence reported for North American postpartum women, which is 13% (95% CI: 11–20%) [37].

In our study, the prevalence of postpartum depression was higher than the prevalence of 24.5% that was previously reported in postpartum women at the same institution using the EDPS questionnaire and the cutoff score of ≥13 to define depression [29]. Based on these results, it is valid to assume that the increase in the prevalence of postpartum depression among these women could be attributable to the COVID-19 lockdown.

Based on the findings reported in a recent systematic review, the prevalence of depression in our study was higher than the overall pooled prevalence of depression (31.4%, 95% CI: 27.3–35.5%) reported in the general population during the COVID-19 lockdown [10]. Our findings are similar to some of the reports of postpartum depression in other countries, such as the United Kingdom [38], Canada [14], Spain [39], and Turkey [18], but the prevalence found in our study was lower than the prevalence of 58% reported among postpartum women in Spain [22]. Likewise, in our study, the prevalence of postpartum depression was higher than the prevalence reported in Italian [40], Chinese [25], and Turkish [41] women. These differences in the prevalence of postpartum depression during the COVID-19 lockdown could be attributed to the ethnic group, differences in the scales used to measure depression, and the postpartum weeks in which they were applied, among other factors.

In our study, the prevalence of trait anxiety in postpartum women was twice that of the non-COVID-19 lockdown period reported in a systematic review, which was 23.4% (95% CI: 13.8–33.0%) [42]. This finding is in line with a recent Chinese study suggesting that prenatal anxiety increases with the severity of the restriction policies [13].

Few studies have reported the prevalence of perceived stress in postpartum women. To the best of our knowledge, this is the first report on perceived stress prevalence during postpartum in Latin-American women during the COVID-19 lockdown. In our study, the mean ± SD of the total score for PSS-10 was similar to that reported in Polish women (18.4 ± 18) [27].

Xiang et al. suggested that the majority of mental health disorders following COVID-19 may be “reactive” in nature, and rather than over-pathologize these experiences, some degree of sadness, anxiety, fear, anger, paranoia, and short-term adjustment issues and long-term adaptation to the uncertain future are perhaps reasonable or expected responses [43]. However, social restrictions, lockdowns, and the reorganization of health systems have been impediments to the face-to-face evaluation of mental health [44], especially in low- and middle-income countries.

A recent systematic review concluded that mental health disorders in pregnant and postpartum women are the outcomes of combined effects, including sociodemographic factors (age, parity, trimester, marital status, educational level, and socioeconomic status), stress (disaster or crisis, life events, marital satisfaction, and medical or obstetric complications), and support from partners, families, societies, and governments) [15]. However, it cannot be inferred that the COVID-19 pandemic is the main reason for the increases in the prevalence rates of mental health disorders [15]. Our study did not find a correlation between most of the sociodemographic factors, except for maternal age, which showed a negative correlation with the total scores for T-STAI and EPDS.

Although a few women with a positive SARS-CoV-2 test were enrolled in our study, there was no correlation between that test results and the total scores of the STAI and PSS-10 questionnaires; however, there was a significant but mild correlation with the EDPS score. There was a lower prevalence of depression, trait anxiety, and perceived stress in the women with a positive SARS-CoV-2 test than those with a negative test, but the difference was not statistically significant. A possible explanation is that most of the SARS-CoV-2 positive women were asymptomatic, and the timing of the infection was 4 to 12 weeks before the women completed the questionnaires such that their mental distress may have either improved or already been resolved.

This study has some limitations: most of the participants had a high-risk pregnancy that was attended in a tertiary level hospital, the wide range of weeks postpartum (4–12) may affect the global prevalence of psychological status, it did not include a nonpregnant comparison group, and the cross-sectional study design generally prevented us from reaching conclusions on the impact of the COVID-19 lockdown on mental health disorders. Additionally, while most of the women who attended our institution were from low- and middle-income families, we did not measure this variable objectively.

Because poor mental health during pregnancy can lead to adverse maternal and infant outcomes, it is important to evaluate the potential influencing factors. In this regard, the risk factors associated with poor mental health include chronic mental illness, somatic illness in the postpartum period, smoking, unplanned pregnancy, and professional status; thus, timely and adequate interventions could improve the short- and long-term outcomes [20].

Postpartum depression affects maternal caregiving in multiple ways, as seen in harmful behaviors, such as substance abuse or self-harm, including suicide. Moreover, depressed women are less likely to breastfeed, attend well-child visits, complete infant immunizations, use safety devices, or place the infant on its back to sleep [21].

A substantial amount of evidence suggests that prenatal and postnatal mental health disorders have a heavy and lasting adverse impact on mothers, fetuses, and children. Mental health disorders were found to be related to socio-emotional, behavioral, and cognitive problems and changes in the brain structures and functions of infants and children [15]. Additional longitudinal studies on both mothers and children are needed to better understand the long-term effects of the COVID-19 lockdown on the mental health of postpartum women.

## 5. Conclusions

The prevalences of depression, anxiety, and perceived stress among postpartum Mexican women during the COVID-19 pandemic lockdown were higher than what has been previously reported in the literature. Opportune and adequate interventions are crucial to improving short- and long-term perinatal outcomes.

## Figures and Tables

**Table 1:** Sociodemographic characteristics of the participants.

Characteristics	Frequency (%) *n* = 293
Age	29.9 ± 6.3 *
Maternal age >35 years	59 (20.1)
Elementary school	18 (6.1)
Middle school	106 (36.1)
High school	104 (35.4)
University and higher	64 (21.8)
Single	83 (28.3)
Married/cohabiting	210 (71.7)
Unemployed	233 (79.5)
Employed	60 (20.5)

* The value is expressed as mean ± standard deviation.

**Table 2:** Characteristics of pregnancy and delivery.

Characteristics	Frequency (%) *n* = 293
Primigravid	96 (32.8)
Chronic hypertension	6 (2.0)
Pregestational diabetes mellitus	13 (4.4)
Positive SARS-CoV-2 test	54 (18.4%)
Preterm birth	40 (13.6)
Preeclampsia	31 (10.6)
Gestational diabetes mellitus	17 (5.8)
Obstetric hemorrhage	10 (3.4)
Obstetric hysterectomy	4 (1.4)
Placenta previa	6 (2.0)
Vaginal delivery/vaginal birth	90 (30.7)
Cesarean	203 (69.3)
Adverse perinatal outcome	101 (34.5)

**Table 3:** Prevalence of depression, trait anxiety, and perceived stress.

Condition	Frequency *n* = 293	Prevalence (95% CI)
Depression	115	39.2 (34–45%)
Trait anxiety	135	46.1 (40–52%)
Perceived stress		
Low	91	31.1 (36–37%)
Moderate	170	58 (52–64%)
High	32	10.9 (7.8–15%)

**Table 4:** Correlation between the total PSS-10 score with the total EPDS and T-STAI scores and the sociodemographic factors.

Variable	PSS-10	T-STAI	EPDS
PSS-10	1	0.835 ***	0.824 ***
T-STAI	0.835 ***	1	0.843 ***
EPDS	0.824 ***	0.843 ***	1
Maternal age (years)	−0.82	−0.121 *	−0.134 *
Number of gestations	−0.008	0.011	0.038
Years of education	0.074	0.063	0.054
Newborn weight (g)	0.066	0.024	0.043
Weeks of gestation at delivery	−0.028	−0.052	−0.093
Live with a partner^1^	0.046	0.057	0.047
Positive SARS-CoV-2^1^ test	0.086	0.101	0.119 *
Adverse perinatal outcome^1^	0.092	0.049	0.027

Pearson and ^1^ Spearman correlation tests: *** *p* < 0.001, * *p* < 0.05.

## Data Availability

The data presented in this study are available upon request from the corresponding author. The data are not publicly available due to privacy.
